# Supplementary material for: A Fine-Tuning of the Plant Hormones, Polyamines and Osmolytes by Ectomycorrhizal Fungi Enhances Drought Tolerance in Pedunculate Oak
Source: Int J Mol Sci. 2023 Apr 19;24(8):7510. doi: 10.3390/ijms24087510 (PMC10139069; doi:10.3390/ijms24087510)
Supplement: Supplementary file 1 [file ijms-24-07510-s001.zip › ijms-2311407-supplementary.pdf]

Table S1. Two-way ANOVA results of variable (ECM, drought and ECM x drought) effects on inspected parameters.

**Parameters**		**ECM**			**Drought**			**ECM x Drought**		
		F	***p< .05***	***p***	***F***	***p< .05***	***p***	***F***	***p< .05***	***p***
**Osmolytes**	Glycine betaine **(GB)**	0.066		7.99e-01	15.115		5.65e-05	5.753		9.00e-03
	Free proline **(PRO)**	11.207		3.00e-03	287.411		1.72e-17	89.113		7.81e-12
	Putrescine **(PUT)**	46.200		4.97e-07	84.796		1.32e-11	40.771		1.91e-08
	Spermine **(SPM)**	0.004		9.52e-01	206.542		7.51e-16	44.898		7.74e-09
	Spermidine **(SPD)**	64.946		2.77e-08	86.937		1.01e-11	54.006		1.30e-09
**Physiological parameters**	Relative water content **(RWC)**	82.26		3.19e-09	21.313		4.77e-06	2.117		1.42e-01
	Net photosynthesis **(A)**	130.735		3.38e-11	102.390		1.78e-12	4.192		2.70e-02
	Transpiration rate **(E)**	122.251		6.67e-11	11.047		3.97e-04	47.177		4.83e-09
	Stomatal conductance **(gs)**	8.733		7.00e-03	34.943		7.79e-08	8.931		1.00e-03
	Water use efficiency **(WUE)**	0.560		4.62e-01	28.313		4.84e-07	10.530		5.21e-04
**Mineral elements**	Nitrogen content **(N)**	2.397		0.135	2.299		0.122	13.035		0.000147
	Carbon content **(C)**	24 2.941		0.099	24 3.559		0.044	2.353		0.117
**Plant hormones**	SA	41.614		1.14e-06	21.271		4.85e-06	47.683		4.36e-09
	JA	1.250		2.75e-01	57.837		6.62e-10	0.762		4.77e-01
	ABA	372.245		4.04e-16	119.786		3.25e-13	106.317		1.18e-12
	OPDA	73.792		8.75e-09	67.431		1.41e-10	52.268		1.80e-09
	SAG	13.212		1.00e-03	26.641		8.05e-07	9.476		9.26e-04
	SGE	69.518		1.51e-08	91.418		5.96e-12	19.284		1.01e-05
	JA.Ile	15.191		0.000682	12.842		0.000161	10.725		0.000470
